# Case Report: Concurrent Resistance and Aerobic Training Regulate Adiponectin Expression and Disease Severity in Multiple Sclerosis: A Case Study

**DOI:** 10.3389/fnins.2020.567302

**Published:** 2020-12-22

**Authors:** Elisa Grazioli, Ersilia Nigro, Claudia Cerulli, Giovanna Borriello, Annamaria Mancini, Eliana Tranchita, Rita Polito, Attilio Parisi, Pasqualina Buono, Aurora Daniele

**Affiliations:** ^1^Department of Exercise, Human and Health Sciences, Foro Italico University of Rome, Rome, Italy; ^2^Department of Environmental, Biological and Pharmaceutical Sciences and Technologies, University of Campania Luigi Vanvitelli, Caserta, Italy; ^3^CEINGE Advanced Biotechnologies, Naples, Italy; ^4^Department of Neurological Sciences, Sapienza University of Rome, Rome, Italy; ^5^Department of Movement, Human and Health Sciences, University of Rome “Foro Italico”, Rome, Italy

**Keywords:** adiponectin, EDSS, HMW oligomers, multiple sclerosis, training

## Abstract

Adapted exercise is an effective non-pharmacological tool to improve functional, cognitive, and psychological parameters in multiple sclerosis (MS), in association with increased quality of life (QoL) and decreased disease severity. Adipose tissue, through the production of different adipokines, is involved in regulating energy metabolism and inflammation. Adiponectin, increased in MS, circulates as oligomers of low (LMW), medium (MMW), and high molecular weight (HMW), the latter mediating the main biological effects. The aim of study was to evaluate the effects of 4 months training at moderate intensity [65% heart rate reserve (HRR)] on BMI, adiponectin, and QoL in a volunteer with secondary progressive MS. The parameters were evaluated before (T0), after 4 months training (T1), and 6 months after the end of training (T2); total serum adiponectin and its oligomeric profile were evaluated. We found a reduction in BMI (−0.9%) and FAT (−2.6%), an improvement in perceived QoL and a reduced expression of total adiponectin and HMW oligomers together with decreased MS disability level at T1 measured by EDSS. Despite the limitations of a case study, this represent a starting point to understand the influence of exercise in MS and the relationship with adiponectin expression.

## Introduction

Multiple sclerosis (MS) is a chronic disabling disease characterized by different clinical phenotypes and whose symptoms may involve different areas, from functional to cognitive or psychological, causing multiple dysfunctions that lead to a progressive worsening of quality of life (QoL) (Thompson et al., 2018). It is estimated that approximately 2.5–3.5 million adults around the world suffer from MS and it is found to be more common in women rather than men (Harbo et al., 2013). A high percentage of MS cases evolves into a secondary progressive form characterized by continuous accumulation of neurological deficits. In the last decade, treatment of relapsing-remitting MS (RRMS) has dramatically improved over the time, although the therapeutic options for secondary progressive MS, both primary and secondary, are still limited (Fitzner and Simons, 2010; Claflin et al., 2018). Regular physical activity not only reduces the risk of developing neurodegenerative diseases such as dementia and Alzheimer disease, but it can ameliorate the prognosis and the symptoms, increasing neurogenesis in the adult brain (Hamer and Chida, 2009; Stephen et al., 2017; Mueller et al., 2019). A recent study supports the positive effects of a regular physical activity on performance, QoL, and fatigue in people with MS (Grazioli et al., 2019b). Physical rehabilitation is reported to be effective in MS patients improving muscle function, balance, walking, movements, and cognitive functions (Centonze et al., 2020). As well known, MS is associated with immune system dysfunctions and chronic inflammation; the induction of inflammation is due to many factors among which the endocrine activity of adipose tissue. In particular, metabolic abnormalities in both cytokines and adipokines levels are one of the mechanisms involved in MS pathogenesis (Gandhi et al., 2010; Mancuso, 2016). Among the others, adiponectin is one the adipokine involved in MS. It has been evidenced that adiponectin seems to impact on immunological function in MS (Rotondi et al., 2013). Adiponectin is the adipokine secreted at highest level in serum where it circulates as oligomers of different molecular weight: low (LMW), medium (MMW), and high (HMW). A previous study on MS patients demonstrated that HMW Adiponectin seems to elicit the biological effects while the other oligomers could differently modulate various signaling pathway (Shehzad et al., 2012). Relevant studies were conducted on adiponectin serum level in MS patients evidencing that, unlike metabolic diseases, systemic autoimmune, and chronic inflammatory diseases are characterized by increased production of adiponectin and HMW oligomers (Piccio et al., 2013; Signoriello et al., 2019). Even if the key role of adipose tissue on both inflammation and immune system was demonstrated, the molecular basis for the serum levels dysregulation as well as the molecular mechanisms played by Adiponectin, are controversial (Devorak et al., 2017). On the other hand, exercise, which induces a reduction in fat mass, could modulate adipokine secretion inducing an insulin sensitizing and anti-inflammatory environment (Negaresh et al., 2018). However, to our knowledge, the studies on the effects of physical activity on inflammatory status in MS patients are rare (Mokhtarzade et al., 2017), although positive effects in other diseases have been clearly described (Beavers et al., 2010; Ferioli et al., 2019). Therefore, the primary outcome of this case-report was to evaluate the impact of a structured physical activity on MS disease progression. To this aim, we analyzed the expression levels of total adiponectin and its oligomerization state in the serum of a 39 years old female MS patient with secondary-progressive MS (EDSS = 4.5), before and after 4 months training program (concurrent aerobic and resistance training). Functional and psychological evaluations were also performed to verify the feasibility and the effects on QoL in the patient. All evaluations were conducted at the baseline (T0), at the end of 4 months training (T1) and 6 months after the end of the program (T2), as follow up, to monitor both the short- and the long-term effects of the combined protocol.

## Case Description

**FIGURE 1:**
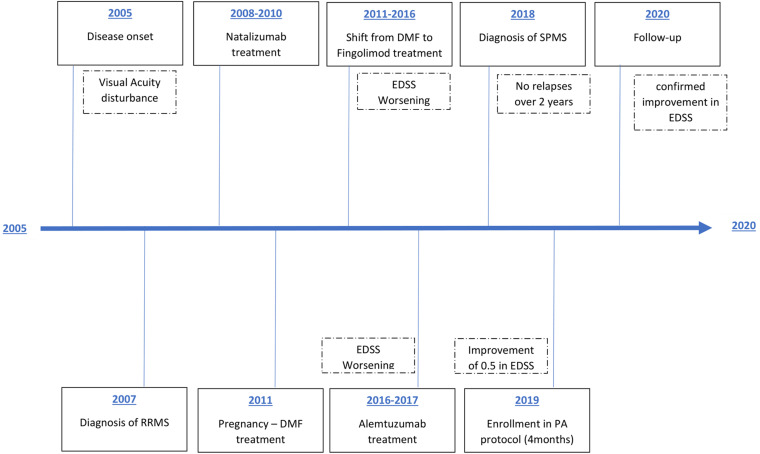
Patient treatment timeline. RRMS, relapsing-remitting multiple sclerosis; DMF, dimethyl fumarate; SPMS, secondary progressive multiple sclerosis; EDSS, Expanded Disability Status Scale.

As it is summarized in Figure 1, after the approval of the ethical committee (RIF.CE: 4520) a 39-year-old with disease onset in 2005 with visual acuity disturbance and diagnosis of RRMS on July 2008, regularly followed in MS Center S. Andrea Hospital, University of Rome Sapienza. She started Natalizumab treatment, stopped after 30 infusions due to de-risking strategy in JCV positivity. After pregnancy on 2011, neurologist opted for treatment with Dimethyl fumarate, stopped after 8 months because persistence of disease activity, so patient was shifted to Fingolimod with partial response and worsening in EDSS score. Considering the young age, Alemtuzumab was started in 2016, with second cycle in February 2017. Unfortunately, patient showed an additional clinical progression, with a stable worsening of 1.5 point in the EDSS at 12 months despite the treatment, configuring a secondary-progressive form of disease. She not experienced relapses at least 1 month before the start of the protocol, the EDSS evaluation was 4.5 at baseline evaluation and she received the authorization to practice Adapted Physical Activity from Sport Medicine Physician at the City University; she was not engaged in physical activity exercise during the 6 months before the beginning of the protocol. All baseline characteristics are reported in Table 1.

**TABLE 1:** Patient characteristics evaluated before the training program.

Patient characteristics	
**Gender**	**Female**
Age (YRS)	39
Height (CM)	173
Weight (KG)	65
BMI	21.7
Type of MS	SPMS
EDSS	4.5

*yrs, years; cm, centimeters; Kg, kilograms, SPSM, secondary progressive multiple sclerosis.*

The primary outcome of the study was to evaluate the effect of a combined training on expression levels of total adiponectin and its oligomerization state. Before (T0), after 4 months of concurrent training (see following) (T1) and 6 months after the end of the protocol (T2) the patient underwent to a functional and psychological (QoL) evaluation as well as a blood sample collection. The second aim was to identify the adiponectin expression and its oligomerization state in a healthy female group and in un untrained MS patient group. Baseline demographic and clinical characteristics of the subject are presented in Table 1. Ten sex- and age- matched healthy controls (all females, mean age 41 ± 2) were enrolled in the study. Ten sex- and age- matched secondary progressive MS patients (all females, mean age 40 ± 3) were recruited. The study was approved by local Ethical Committee, all patients gave their informed consent to participate in the study.

### Anthropometric Measurement

The assessment of body composition (fat mass percentage (FAT %), and free fat mass (FFM %) was obtained using the digital bioelectrical impedance device (Handy 3000; DS Medica, Milan, Italy), with a frequency of 50 and 100 kHz. Body mass index (BMI) was also determined. In order to make the measurement reproducible, the patient has to be fasting for 4 h, no physical activity for 12 h, no alcohol, and diuretics (unless prescribed) for 48 h, well hydrated (water only).

#### Physical Performance Assessment

The 6-min walking test (6MWT) was used to assess endurance and functional capacity (Goldman et al., 2008); the Handgrip test was performed to measure the muscular strength through the handgrip dynamometer (Jamar Plus^®^; Patterson Medical Ltd.) for each arm (Hamilton et al., 1992); and the Timed Up and Go Test (TUG) to evaluate the muscular function and mobility. The National Institute of Clinical Evidence (NICE) guidelines also advocate the use the TUG test for assessment of gait and balance in the prevention of falls (Kang et al., 2017).

#### Questionnaire

The Multiple Sclerosis Quality of Life-54 (MSQOL-54) (Solari et al., 1999), which investigate physical functions, limitations related to the physical and emotional sphere, perception about health, social, cognitive and sexual functions, and general QoL. The MSQOL-54 summary scores are the physical health composite summary and the mental health composite summary. The Fatigue Severity Scale (FSS) it is designed to differentiate fatigue from clinical depression in these patients and to explore severity of fatigue symptoms (Ottonello et al., 2016). The PHQ-9 Questionnaire used as a screening tool for major depressive disorder (Patrick and Connick, 2019).

### Adiponectin Evaluation

#### Blood Sampling and Analysis

A total of 24 h before training and follow final training session, a venous blood sample (10 ml) was collected after a 10-h overnight fast between 7:30 a.m. and 9:00 a.m. Total serum adiponectin of healthy controls, MS patients, and the MS case undergone a physical activity training was measured by ELISA-test using house-produced polyclonal antibodies as previously described (Daniele et al., 2012). Each serum sample was tested three times in triplicate.

#### Western Blotting Analysis

Serum proteins were isolated from healthy controls, MS patients, and the MS case undergone a physical activity training as previously described by Signoriello et al. (2019). For the immunoblot analysis, an equal amount of proteins (10 μg) was resolved in SDS-polyacrylamide (BIO-RAD) gels (10%) and transferred onto nitrocellulose membranes (Amersham). Thereafter, membranes were incubated with adiponectin antibodies (Novus Biologicals, Littleton, CO, United States) appropriately diluted in Tween Tris–buffered saline (TTBS). Proteins were revealed by the enhanced chemiluminescence (ECL) with Kodak BioMax Light film, (GE Healthcare Bio-Sciences Pittsburgh, PA, United States), digitalized with a scanner (1200 dpi) and analyzed by densitometry with ImageJ Software^1^. All samples were tested twice in duplicate.

After 6 months, during which the patient was not enrolled into a structured physical activity protocol, the same evaluations were performed to verify the long-term effect of the concurrent training, and the IPAQ questionnaire was administered in order to assess the total level of physical activity during the follow up.

### Concurrent Resistance and Aerobic Training

Adapted concurrent training and 1-RM assessment: The patient was enrolled in a structured concurrent training, which last 4 months (34 sessions, twice a week, 50 min per session). The single session of this protocol was divided in four phases: warm-up, resistance training, endurance training, and cool down. The first phase of warm up consisted in 3 min of stationary bike without slope and resistance and 2 min of dynamic warm up for the upper body. In the second phase the patient was involved in strength exercises, where the loads of the upper and lower limbs were set at 50% of 1-RM of the patient, using six types of strength machines (Technogym equipment): Leg Press, Leg Extension, Leg Curl, Vertical Traction, Row Pull, and Abdominal Crunches. After a period of familiarization, the maximal strength was assessed by one repetition maximum (1-RM) test using Brzycki equation (Abdul-Hameed et al., 2012). The same equipment used for the 1-RM assessment were used for the resistance training that lasted 25 min and consist in eight repetitions for two sets, with 1 min of rest between the sets. The third phase consisted in aerobic training including 10 min of stationary bike at moderate intensity, 65% heart rate reserve (HRR) evaluated using the Karvonen formula: Exercise HR = % of target intensity (HRmax – HRrest) + HRrest (Hansen et al., 2012). The last phase consisted in 10 min of cool down involved static stretching for all major muscle trained: shoulders and upper limbs, trunk and lower body, both in sit and in stand position.

## Outcomes

**FIGURE 2:**
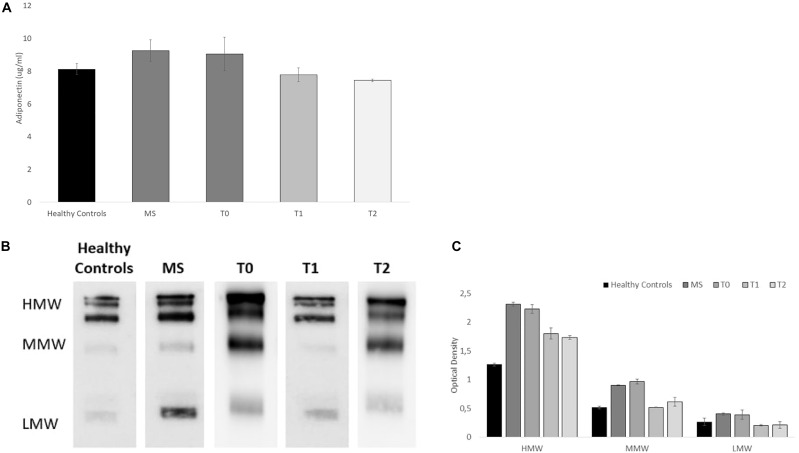
Adiponectin serum levels and Serum Acrp30 distribution in healthy controls (n = 10), MS patients (*n* = 10), and in the MS case undergone a training program at baseline (T0), post physical activity intervention (T1), and at the follow-up (T2): **(A)** Elisa-test of Adiponectin serum level (μg/ml). The values are reported as mean of the absorbance ± SD; **(B)** One representative image of western blot oligomeric distribution [HMW (≥250 kDa), MMW (180 kDa), and LMW (70 kDa)]; **(C)** Graphical representation of pixel quantization of HMW, MMW, and LMW oligomers. For other details see section “outcomes.”

As shown in the Elisa-test (see Figure 2A), we analyzed total serum adiponectin in healthy controls, MS patients, and baseline (T0), post physical activity intervention (T1) and at follow-up (T2) in the MS case patient, in which, we found a decrease of serum adiponectin levels [9.05 μg/ml (T0) vs. 7.78 μg/ml (T1)] before and after physical activity intervention. Interestingly, at the follow-up (T2), adiponectin levels remained stable (7.44 μg/ml) indicating that the effects of the training last for at least 6 months. As control, we confirmed that higher levels of serum adiponectin were found in untrained MS patient compared to healthy controls (9.25 vs. 8.13 μg/ml, respectively, *p* < 0.05).

Furthermore, as shown in the Western blots (Figures 2B,C), we analyzed the distribution of serum Acrp30 in healthy controls, MS patients, and baseline (T0), post physical activity intervention (T1) and at follow-up (T2) in the MS case patient. Three bands corresponding to HMW (≥250 kDa), MMW (180 kDa), and LMW (70 kDa) oligomers were evident in MS patient (Figure 2B). The densitometric evaluation of oligomeric distribution showed that the expression of HMW, MMW, and LMW oligomers are higher in MS patients compared to healthy controls (*p* < 0.05). When we analyzed adiponectin oligomeric profile in the MS case undergone physical activity, we found that all oligomers are lower in the MS patient after physical activity intervention compared to baseline. In particular, a more evident decrease in HMW oligomers, the most biologically active oligomers, was found (Figure 2C). The adiponectin oligomerization state at the follow-up (T2), do not substantially change for all oligomers (Figure 2).

### Functional and Psychological Outcomes

As shown in Table 2, all functional parameters improved after 4 months of concurrent aerobic and resistance training. The patient reported a decreased BMI (−0.9%), more in deep the impedance analysis showed an increase of FFM (+0.8%) and a decrease of FAT (−2.6%) suggesting a positive modification of body composition. The tests related to walking ability and autonomy showed an improvement at T1 (10mwt = −4.3%; 6mwt = 6.7%), as well as the strength (Handgrip Sx = 8.9%; Handgrip Sx = +23%) and flexibility (TR Dx = +37%; TR Sx = 106.1%) analysis, confirming the functional improvements on these patients due to an adapted exercise. The TUG test performance reported a slight decrease after the protocol (+1.6%), instead the Berg Balance score did not reveal changes over the time, probably due to the good starting point of the patient. After 6 months, from the end of the protocol, most of the evaluated parameters decrease returning to the basal level. As shown in Table 2, BMI increase at T2 compare to T1 and T0 (T1/T2 = +2.8%; T0/T2 = 1.8%), the body composition analysis revealed that FFM decrease (T1/T2 = −2.0%; T0/T2 = −1.2%), and FAT increase (T1/T2 = +13.2%; T0/T2 = +9.4%). The tests related to walking ability and autonomy reported a worsening in these parameters after 6 months (T2), compared to T1 and T0 (TUG T0/T2 = +1%; 10mwt T1/T2 = +4.5%; 10mwt T0/T2 = 0%). An exception was reported by the TUG analysis between T1 and T2 (−0.6%) and the 6mwt that did not showed changes at T2, both comparing to the data collected at T0 and T1. The IPAQ at T2 revealed a LOW level meaning that the volunteer did not reach the criteria for either moderate or high levels of physical activity. The EDDS evaluation showed a decrease after training (EDDS T0/T1 = −11.1%) that remain the same after 6 months of follow up, suggesting the general positive effect of this protocol on the disease severity and on patient functionality.

**TABLE 2:** T0, T1, and T2 functional and psychological parameter evaluations.

Functional and psychological parameters	T0	T1	T2	% T0/T1	% T1/T2	% T0/T2
BMI	21.7	21.5	22.1	−0.9	+2.8	+1.8
FFM (KG)	49.5	49.9	48.9	+0.8	−2.0	−1.2
FAT (KG)	15.5	15.1	17.1	−2.6	+13.2	+9.4
TUG (SEC)	5.5	5.6	5.5	+1.6	−0.6	+1.0
10MWT (SEC)	3.5	3.3	3.5	−4.3	+4.5	0
TR DX (CM)	57.0	78.5	54.0	+37.7	−31.2	−5.6
TR SX (CM)	41.0	84.5	40.0	+106.1	−52.6	−2.5
HG DX (CM)	16.8	18.3	17.9	+8.9	−2.2	+6.1
HG SX (CM)	19.1	23.5	18.9	+23.0	−20.4	−2.1
6MWT (MT)	525	560	525	6.7	0	0
EDSS	4.5	4	4	−11%	0%	−11%
BERG (PT)	56	56	56	0	0	0
45 MSQOL P	46.8	49.2	47.1	+5.3	−4.3	+0.6
45 MSQOL M	43.9	48.8	32.7	+11.2	−12.5	−2.8
FSS	5.6	5.3	5.0	−5.4	−5.7	−12
PHQ	10.0	6.0	5.0	−40.0	−16.7	−100

*Differences between T0 and T1, T0 and T2, and T1 and T2 is expressed by %. BMI, body mass index; FFM, free fat mass; FAT, fat mass; TUG, timed up and go; 10mwt, 10 meter walking test; TR Dx, trunk rotation right; TR Sx, trunk rotation left; HG Dx, handgrip right; HG Sx, handgrip left; 6mwt, 6-min walking test; EDSS, Expanded Disability Status Scale; BERG, Berg Balance Scale; 45MSQOL P, physical health composite score of multiple sclerosis quality of life-54; 45MSQOL M, mental health composite score of multiple sclerosis quality of life-54; FSS, Fatigue Severity Scale; PHQ, patient health questionnaire.*

The psychological parameters (Table 2) reported an increase in QoL, both in physical (45MSQOL P T0/T1 = +5.3%) and mental (45MSQOL M T0/T1 = +11.2%) Score after the concurrent training, the patient experienced a general well-being after the protocol, she also revealed a decrease in Fatigue perception (FFS T0/T1 = −5.4%) and Depressive status (PHQ T0/T1 = −40%) at T1. According to the results Fatigue and Depression have decreased further after 6 months (FFS T1/T2 = −5.7; PHQ = −16.7) and the scores did not return to the baseline level over the time (FFS T0/T2 = −12%; PHQ T0/T2 = −100%), suggesting that the effect of these training could be a long term effect. The QoL, instead, decreased after 6 months compare to T1 (45MSQOL P T1/T2 = −4.3%; 45MSQOL M T1/T2 = −12.5%) and almost return to the baseline level (45MSQOL P T0/T2 = +0.6%; 45MSQOL M T0/T2 = −2.8%).

## Discussion

The results of this case report suggest that a well-structured and adapted concurrent aerobic and resistance training can decrease the Adiponectin levels, as well as the HWM oligomers already after 4 months of training. The study also confirms the feasibility of the training and evidenced the positive effects on functional and psychological parameters, increasing the QoL. This is the first study highlights a correlation between physical activity, adipokines level, and inflammation in MS. Indeed the follow up analysis (T2) showed that the adiponectin levels do not return to baseline level after 6 months but remain similar to those found after the training period (T1). Despite adiponectin expression seems to be independent from body composition changes in MS patients, an increased FAT, and a decreased FFM can influence the disease severity and progression (Ben-Zacharia, 2018; Pilutti and Motl, 2019). Interestingly, the same trend found in adiponectin levels was detected by the analysis of the functional and psychological data. This could further suggest that the decrease in adiponectin level might be related to an increase of several functional and psychological parameters after the concurrent aerobic and resistance training and at 6 months follow up in a woman affected by a secondary progressive MS. Previously, conflicting with our results, Mokhtarzade et al., 2017 found an adiponectin level increase in MS women after exercise training (Mokhtarzade et al., 2017). However, a possible explanation for these different results could be ascribed to the different time and type of exercise intervention and/or to different clinical and metabolic features of the MS patients analyzed (Mokhtarzade et al., 2017). Indeed, MS patients considered in the paper by Mokhtarzade et al. (2017) have a relapsing-remitting phenotype while the MS patient considered in the present paper is a secondary-progressive one. Previous studies correlated adiponectin levels and MS disability evaluated by the EDSS score (Çoban et al., 2017; Signoriello et al., 2018); it is plausible that adiponectin modulation is due to its involvement in the chronic inflammatory processes as well as in immune dysregulation, both processes typical of MS. In fact, MS is characterized by aberrant peripheral immune cells invading the CNS that induce further inflammation and create a complex network of immune cells and inflammatory factors (Zhou et al., 2020). On the other hand, beyond its metabolic functions, an immune modulatory and anti-inflammatory role for adiponectin has been highlighted in several immune disorders (Pecoraro et al., 2017; Signoriello et al., 2018). Targeting the immune system under physiological and pathological conditions, adiponectin acts on diverse cells resulting in immune suppression and anti-inflammatory response (Luo and Liu, 2016). The same inflammatory mechanisms could be influenced by exercise, which can exert a great impact on MS disability level as recently suggested by Afzal et al. (2020). Our data support this hypothesis indeed we observed a decrease in the EDSS score following 4 months of training. Indeed, our MS patient had an EDSS score of 4.5 at baseline and reported a decrease of 0.5 after 4 months of training, which do not increase after 6 months of follow-up. Growing evidence has shown the beneficial influence of exercise on humans (Liu et al., 2019; Rizzo et al., 2020); besides cardiometabolic positive effects, physical activity acts also on brain health, reducing the risk of neurodegenerative diseases as dementia (Hamer and Chida, 2009; Liu et al., 2019). In MS, physical activity not only reduces the incidence of the disease but has also disease-modifying effects (Dalgas et al., 2019; Grazioli et al., 2019b) and our data seems to confirm that exercise decreases clinical severity.

## Conclusion

Recently, Marck et al., suggested that people with more severe symptoms and progressive disease, as our case is, may require focused exercise promotion to achieve beneficial results. Our study presents some limitations such as the lack of genetic background of the case patient; however, the relevance of the study is represented by the characterization of adiponectin profile that highlights the increase of HMW, the oligomers reported as the most biological isoforms of this adipokine. Then, although these are preliminary data, this study implements the evidence that exercise could be a valid therapy in secondary progressive MS and that adiponectin seems to be a potential clinical marker able to predict the disease progression (Okada-Iwabu et al., 2015; Çoban et al., 2017). Further studies are needed to clarify if body composition and exercise can affect the mechanisms involved in adiponectin expression and MS severity and progression.

## Data Availability Statement

The raw data supporting the conclusions of this article will be made available by the authors, without undue reservation.

## Ethics Statement

The studies involving human participants were reviewed and approved by the Comitato Etico dell’Università Sapienza – Rome, Italy (RIF.CE: 4520). The patients/participants provided their written informed consent to participate in this study. Written informed consent was obtained from the individual(s) for the publication of any potentially identifiable images or data included in this article.

## Author Contributions

AP, PB, and AD contributed to conception and design of the study. EG and CC organized the methodology. ET and GB performed the clinical analysis. AM and RP performed the biochemical analysis. EG wrote the first draft of the manuscript. PB, AD, AM, and RP wrote the Adiponectin evaluation and results sections. All authors contributed to manuscript revision, read, and approved the submitted version.

## Conflict of Interest

The authors declare that the research was conducted in the absence of any commercial or financial relationships that could be construed as a potential conflict of interest.

## Footnotes

1 https://imagej.nih.gov/ij/download.html
